# Draft Genome Sequence of *Alkalinema* sp. Strain CACIAM 70d, a Cyanobacterium Isolated from an Amazonian Freshwater Environment

**DOI:** 10.1128/genomeA.00635-17

**Published:** 2017-07-13

**Authors:** Alex Ranieri Jerônimo Lima, Wendel de Oliveira Castro, Pablo Henrique Gonçalves Moraes, Andrei Santos Siqueira, Délia Cristina Figueira Aguiar, Clayton Pereira Silva de Lima, João Lídio Silva Gonçalves Vianez-Júnior, Márcio Roberto Teixeira Nunes, Leonardo Teixeira Dall’Agnol, Evonnildo Costa Gonçalves

**Affiliations:** a Laboratório de Tecnologia Biomolecular, Instituto de Ciências Biológicas (ICB), Universidade Federal do Pará (UFPA), Belém, Pará, Brazil; b Laboratório de Farmacognosia, Programa de Pós-Graduação em Saúde e Ambiente (PPGSA), Universidade Federal do Maranhão (UFMA), São Luís, Maranhão, Brazil; c Centro de Inovações Tecnológicas (CIT), Instituto Evandro Chagas (IEC), Ananindeua, Pará, Brazil; d Universidade Federal do Maranhão (UFMA), Campus de Bacabal, Maranhão, Brazil

## Abstract

In order to increase the genomic data of cyanobacterial strains isolated in Brazil, we hereby present the draft genome sequence of the *Alkalinema* sp. strain CACIAM 70d, isolated from an Amazonian freshwater environment. This report describes the first genome available for this genus.

## GENOME ANNOUNCEMENT

*Cyanobacteria* (domain *Bacteria*) is one of the oldest and most morphologically diverse phyla on the planet (1). Nowadays, this diversity has been characterized by a polyphasic approach, which has resulted in the identification of a number of new taxa (2). Based on this approach, Vieira Vaz et al. (3) described *Alkalinema*, a novel cyanobacterial genus, whose representatives are morphologically similar to *Leptolyngbya*. So far, *Alkalinema* was found only in one saline-alkaline lake from the Brazilian Pantanal wetlands (3). Thus, to the best of our knowledge, there is little information about this genus, especially with regard to molecular data.

Here we recovered, from a nonaxenic culture, the draft genome sequence of a cyanobacterial strain which was isolated from the Tucuruí hydroelectric power station reservoir (3°50′04.9″S, 49°42′32.2″W) in Pará, Brazil. A Bayesian inference based on the 16S rRNA gene of this strain (data not shown) indicated its close phylogenetic relationship to genus *Alkalinema*, which was then identified as *Alkalinema* sp. strain CACIAM 70d.

BG-11 medium was used to measure biomass increase. Total DNA was obtained using a modified phenol-chloroform-based protocol (4). One sequencing run was performed on a GS FLX 454 platform using a nonpaired library. The raw reads obtained were quality filtered (minimum Phred score, 20) and then assembled using Newbler version 2.9 (parameters, minimum overlap of 40 bp, minimum overlap identity of 90%, heterozygote mode, and extended low-depth overlap options). MaxBin 2.2.1 (5) was used to bin the assembled contigs. To classify taxonomically the obtained bins, we performed BLASTp (6) for each bin in the sequences containing Hidden Markov Models for essential genes identified by MaxBin 2.2.1 against the NCBI nonredundant database. The results were visualized on MEGAN 5 (7).

Under this metagenomic approach, it was possible to recover the draft genome of *Alkalinema* sp. CACIAM 70d, which has 6.4 Mb, 223 contigs (ranging from 1,000 to 325,614 bp), an *N*_50_ of 82,256 bp, and G+C content of 49.6%. The genome structural annotation was carried out using the NCBI PGAP (8), which identified 5,379 coding sequences (CDSs), 71 tRNAs, 4 rRNAs, 3 noncoding RNAs (ncRNAs), and 2 transfer-messenger RNAs (tmRNAs). The genome of *Alkalinema* sp. CACIAM 70d was estimated by MaxBin 2.2.1 to be 97.2% complete.

Preliminary analysis with antiSMASH 3.0 (9) revealed the presence of unknown bacteriocin and lantipeptide gene clusters, as well as nonribosomal peptide synthetase-polyketide synthase (NRPS-PKS) gene clusters showing low similarity with curacin and naphthoxanthene antibiotic FD-594 clusters. Furthermore, a COG analysis (10) pointed out 91 genes present in the category “Secondary metabolites biosynthesis, transport, and catabolism.”

This report can improve the genomic data about the genus *Alkalinema*, and the overall *Cyanobacteria* group, by including the first genome sequence available for this genus.

### Accession number(s).

This whole-genome shotgun project has been deposited at DDBJ/EMBL/GenBank under the accession number MUGG00000000. The version described in this paper is version MUGG01000000.
